# Exercise: A Gate That Primes the Brain to Perform

**DOI:** 10.3390/brainsci10120980

**Published:** 2020-12-14

**Authors:** Stéphane Perrey

**Affiliations:** EuroMov Digital Health in Motion, Univ Montpellier, IMT Mines Ales, 34090 Montpellier, France; stephane.perrey@umontpellier.fr; Tel.: +33-(0)4-34-43-26-23

The improvement of exercise performance encountered in sports not only represents the enhancement of physical strength but also includes the development of psychological and cognitive functions. Accumulating evidence has showed that physical exercise is a powerful way to improve a number of aspects of cognition and brain functions at the system and behavioral levels. Yet, several questions remain: what type of exercise program is the most optimal for improving cognitive functions? What are the real effects of some innovative exercise protocols on the relationship between behavior and the brain? To what extent do ergogenic aids boost cognitive functions? What about the efficacy of neuromodulation techniques on behavioral performance? Answers likely require combined insights not only from physiologists and sports scientists, but also from neuroscientists and psychologists. Published manuscripts (sixteen research papers and one perspective article from various academic fields) within this Special Issue “Studying Brain Activity in Sports Performance” did bring novel knowledge and new directions in human exercise-cognition research dealing with performance. Here, we summarize the main insights provided by the contributions and showcase the multiple relationships between cognitive functions, brain activity, and behavioral performance with applications in sports and exercise science.

First, the cognitive benefits of acute and chronic (training) physical exercise need to move beyond simple aerobic activities and resistance training. From a multidisciplinary perspective, this Special Issue proposes some encouraging evidence for other types of physical exercise (more demanding/challenging) or interventions (ergogenic help), improving executive functions for various populations (children to older adults, in healthy and diseased states) that have received far less attention. Following acute endurance exercise of 30 min at constant moderate intensity on a cycle ergometer, Wang et al. [1] observed noticeable facilitation effects on the formation of long-term declarative memory and procedural memory functions that are essential for the development of motor skills, for instance. This study highlights for the first time that traditional endurance-type exercise impacts memory processing by facilitating the encoding period of declarative memory and the consolidation period of procedural memory. Regarding endurance training, it is thought that high-intensity intermittent training consisting of alternating periods of intensive aerobic exercise with periods of recovery may be considerably more effective at improving cognitive function. The effects of such exercise intensity changes on executive functions were examined by Mekari et al. [2]. Their original findings showed that specific executive functions (e.g., flexibility) were more sensitive to high intensity interval training as compared to regular endurance training at moderate intensity in young adults after 6 weeks (3 days/week). Another common exercise modality, resistance exercise (integrated in weight training), is also recognized to improve brain function (see, for a review, [3]). Resistance exercise involves the voluntary activation of specific skeletal muscles against some form of external resistance, provided by body mass, free weights, or a variety of exercise equipment including machines, elastic bands, or manual resistance. The study of Wilke et al. [4] provided new outcomes of interest for such approaches. As compared to machine-based training, the use of free weights appears to be a more effective resistance exercise method to acutely improve cognitive function (i.e., increasing inhibitory control) than the use of conventional training machines in healthy adults [4]. The authors suggested that engagement in free-weight exercise is likely linked to more complex cortical activation patterns when compared to machine-based resistance training or aerobic exercise. While a large body of evidence is available for endurance and resistance training protocols, Wilke and Royé [5] proposed to investigate the beneficial effects of high-intensity functional training that concurrently integrates cardiovascular (endurance) and muscular (resistance) exercises. Through a three-armed randomized crossover trial in healthy adults, exercise intensity did not substantially alter short-term lower-order executive functions, such as attention, working memory, inhibitory control, and cognitive flexibility. However, these preliminary findings indicate that the highest exercise intensities in functional circuit training might be more advantageous to enhance in concert cognitive, cardiovascular, and muscular functions.

Besides healthy adults, a few original articles in this Special Issue were related to other target clinical populations and aimed to test the beneficial effects of novel exercise protocols on cognition. Wen and Tsai [6] investigated the effects of a 30-min bout of supervised moderate-intensity aerobic (dance) combined with resistance exercise on neurophysiological (i.e., behavioral and cognitive electrophysiological) performance in sedentary obese female adults with impaired neurocognitive functions. The authors observed no behavioral (inhibitory control) benefits by the exercise mode but reported improved brain neural processing related to early and late inhibition, captured by brain electrical activity (e.g., event-related potential recording provided by electroencephalography—EEG). In children aged 3–6 years with autism spectrum disorder, Wang et al. [7] showed that implementation of cognitively and/or coordinatively demanding physical exercises (mini-basketball) significantly improved all aspects of executive functions, including working memory and inhibition, following a 12-week training program, as compared to the control group. Beyond these first behavioral outcomes in most of the aforementioned studies, neural correlates (e.g., changes in functional brain activity patterns) of the observed cognitive changes need to be further investigated as carried out by Wen and Tsai [6]. In elite basketball players, Chiu et al. [8] investigated both behavioral responses but also neural correlates that modulated the executive functions as a function of playing positions in basketball. This study showed divergent neural processing efficiency based on EEG recordings related to player position (guards were more efficient than forwards) when performing a cognitive task involving inhibitory control. This suggests that cognitive functioning in open-skilled sports might be dependent on the player’s position.

Executive functioning is important for athletic performance and can be affected by ergogenic aids. The ergogenic effect of caffeine might not only enhance physical strength but also the development of cognitive functions during exercise. Wang et al. [9] in a cross-over double-blind study showed that ingestion of a low dose of caffeine had greater positive effects on intermittent exercise (mimicking team sports) and cognitive performance (perceptual-motor speed, executive information processing) than a moderate or high dose of caffeine. In a double-controlled experimental design study, Brietzke et al. [10] showed that a carbohydrate mouth rinse might counteract mental fatigue effects on exercise performance, despite comparable cortical activation assessed by functional near-infrared spectroscopy (fNIRS), an optical neuroimaging method that allows for measuring brain tissue concentration changes in oxygenated and deoxygenated hemoglobin in the cortical layers during exercise [11]. The benefit of applying functional neuroimaging methods, such as fNIRS or EEG, in the field of exercise-cognition research, allows the investigation of to what extent exercise-induced changes in neural processes underlie behavioral outcomes; these methods are presenting a growing interest [10,11,12,13]. The study of Brietzke et al. [10] provided an example of a rigorous, well-controlled methodology to assess EEG responses over the prefrontal cortex and primary motor cortex areas at different controlled intensities below 75% of the maximal power output during a maximal incremental cycling test. 

Second, after establishing a link between exercise and the outcomes of some specific cognitive function tests, using neuroimaging techniques is required to reveal the electrophysiological (EEG) processes that are associated with some behavioral changes, or evaluate the physiological changes (e.g., increased cerebral blood flow and cerebral metabolism with fNIRS) over the course of the intervention or training program, that have the potential to improve executive functions. In their perspective article, Herold et al. [11] overviewed the most current and suitable portable neuroimaging methods to monitor brain activity during physical exercise. The most common methods used to investigate effects on functional brain activation are fNIRS and EEG. Herold et al. [11] emphasized that brain activity derived from fNIRS measures could be used as valuable and promising indicators of internal load (e.g., cognitive load and fatigue, stress, traits) during physical exercise. Prescribing exercise intensity by using such neurocognitive outcomes sensitive to psychophysiological responses is clearly lacking in sport and exercise sciences and clinical settings. Monitoring cortical hemodynamics opens up a new perspective on exercise–cognition interaction but needs to be further evaluated. The study from Stute et al. [12] advanced knowledge about acute exercise effects from a neurocognitive perspective by assessing cortical hemodynamic activity in the frontal and parietal cortices during cognitive testing before and at three time points post exercise (i.e., 15, 30, and 45 min) in a sample of healthy older adults. The authors concluded that exercise might somewhat influence working memory performance for up to 45 min due to a more heightened executive processing and attention component of working memory in the frontal cortex. Most sports are self-control demanding, such as during a sprint running start. Using fNIRS to monitor cortical hemodynamic changes during a sprint start sequence through a randomized within-subject design in 60 young adults, Stadler et al. [13] provided new support for the involvement of the ventrolateral prefrontal cortex after the set signal, while highlighting gender differences in the processing of sprint start-induced self-control demands. Still, according to a psychological framework, volitional quality as a mental trait for dealing with adverse circumstances is often met in sports. In a parcel-wise brain morphometry study by comparing elite athletes (short track speed skate, national team) with controls, Wei et al. [14] observed that long-term sports training might improve the mental characteristics of volitional qualities. Indeed, they identified specific cortical architecture (e.g., a greater cortical thickness in the left inferior parietal lobule) associated with volitional qualities. Finally, it is now well acknowledged that physical performance is limited by the perception of the effort a task induces and not only by the limits of the physiological systems. Emerging evidence suggests that the maintenance of ongoing physical exercise requires mental effort. Aiming to ascribe a key role to the psychological concept of self-control in the effective regulation of physical performance, Giboin et al. [15] showed according to their experimental design (income manipulation) a more efficient use of resources while performing a strenuous lower-limb isometric task, allowing for a longer time before self-disengagement without a change in psychological (rating of perceived exertion), cortical (lateral prefrontal cortex), or physiological (neuromuscular fatigue) markers of effort. It is possible that some prefrontal-dependent functions are down-regulated to save the mental effort and executive resources that are needed to maintain the physical task.

Third, enabling enhanced physical exercise may benefit from muscle strength and whole-body movement improvements. Within this context, non-invasive brain stimulation (e.g., neuromodulatory techniques such as transcranial direct current stimulation, tDCS) approaches have been employed to uncover strength-related brain–muscle (movement) associations. By applying tDCS over the primary motor cortex and the cerebellum with traditional montages, a randomized counter-balanced sham-controlled double-blinded cross-over design study from Kenville et al. [16] provided novel finding that anodal cerebellar tDCS can improve static force output during whole-body movement production regularly used in resistance training. Two other pilot studies in this Special Issue provided further critical knowledge on the absence of evidence of the effects of high-definition tDCS (HD-tDCS) on functional performance parameters of the foot [17] and wrist [18]. In a randomized double-blinded, self-controlled study, Xiao et al. [17] examined, in young adults, the effects of a single-session anodal HD-tDCS designed to target the sensory-motor regions of the brain with respect to foot muscle strength, passive ankle kinesthesia, and static balance. In a double-blind sham-controlled study, Besson et al. [18] tested the hypothesis that multiple sessions of cathodal priming and anodal tDCS over 3 consecutive days could further enhance motor learning and retention of an already learned visuomotor task compared to anodal tDCS or sham. While both studies observed some positive changes, no significant differences were observed between HD-tDCS and sham stimulation conditions, likely due to ceiling effects in healthy participants. 

To conclude, this Special Issue showed that the acute or chronic exercise effects upon cognitive functions are varied and dependent upon exercise duration, intensity, and mode, as well as the type of cognitive tasks assessed. The considerable advancement in wearable neuroimaging methods coupled with neural recording and non-invasive brain stimulation for studying human brain physiology are now enabling the gaining of new insight into the complexities of the behavior–exercise relationship, where the prefrontal cortex as a whole plays a cardinal role in the temporal organization of behavior and cognitive activities. This Special Issue makes the point that exercise and sport sciences are a joint research initiative of exercise scientists, psychologists, neuroscientists, neurophysiologists, and other related disciplines such as neuroimaging.
